# PET-CT Imaging in Hypertrophic Cardiomyopathy: A Narrative Review on Risk Stratification and Prognosis

**DOI:** 10.3390/diagnostics15020133

**Published:** 2025-01-08

**Authors:** Patrícia Marques-Alves, Lino Gonçalves, Maria João Ferreira

**Affiliations:** 1 Cardiology Department, Coimbra Hospital and University Center, 3004-561 Coimbra, Portugal; 2 Faculty of Medicine, University of Coimbra, 3004-548 Coimbra, Portugal; 3 Instituto Ciências Nucleares Aplicadas à Saúde (ICNAS), 3000-548 Coimbra, Portugal; 4 Coimbra Institute for Biomedical Imaging and Translational Research (CIBIT), 3000-548 Coimbra, Portugal

**Keywords:** hypertrophic cardiomyopathy, PET-CT imaging, sudden cardiac death

## Abstract

Hypertrophic cardiomyopathy (HCM) is a heterogeneous cardiac disease and one of its major challenges is the limited accuracy in stratifying the risk of sudden cardiac death (SCD). Positron emission tomography (PET), through the evaluation of myocardial blood flow (MBF) and metabolism using fluorodeoxyglucose (FDG) uptake, can reveal microvascular dysfunction, ischemia, and increased metabolic demands in the hypertrophied myocardium. These abnormalities are linked to several factors influencing disease progression, including arrhythmia development, ventricular dilation, and myocardial fibrosis. Fibroblast activation can also be evaluated using PET imaging, providing further insights into early-stage myocardial fibrosis. Conflicting findings underscore the need for further research into PET’s role in risk stratification for HCM. If PET can establish a connection between parameters such as abnormal MBF or increased FDG uptake and SCD risk, it could enhance predictive accuracy. Additionally, PET holds significant potential for monitoring therapeutic outcomes. The aim of this review is to provide a comprehensive overview of the most significant data on disease progression, risk stratification, and prognosis in patients with HCM using cardiac PET-CT imaging.

## 1. Introduction

Hypertrophic cardiomyopathy (HCM) is a heterogeneous cardiac disorder caused by autosomal dominant mutations in sarcomeric genes, leading to a wide range of phenotypic expressions. Clinically, some patients remain asymptomatic throughout their lives, while others develop symptoms such as angina, syncope, or heart failure [1,2,3]. The mortality rates associated with hypertrophic cardiomyopathy (HCM) have significantly declined over time. Earlier studies reported rates as high as 6% per year in hospital-based cohorts, but these are now recognized as overestimates due to the preferential referral of higher-risk patients to specialized tertiary centers [4]. More representative data from less selective cohorts have indicated HCM-related sudden death (SCD) mortality rates of approximately 1.5% per year [4]. With the widespread adoption of implantable cardioverter defibrillators (ICDs) in the HCM population, this rate has further decreased to about 0.5% per year [5,6,7].

One of the major challenges in managing HCM is the limited accuracy of current risk stratification methods for predicting SCD [6,8]. ICDs are the only established preventive therapy, but this creates a difficult balance for clinicians who aim to prevent SCD while minimizing the burden of invasive therapies.

The HCM Risk-SCD score is based on maximal wall thickness, left atrial diameter, maximal left ventricular outflow tract gradient, family history of SCD, presence of non-sustained ventricular tachycardia, and unexplained syncope. It should not be used in competitive athletes and metabolic diseases and it has not been validated following myectomy or septal ablation [2,3,9]. The traditional clinical profile of patients with HCM at the highest risk of SCD primarily includes young individuals, particularly those under 30 years old, such as children and adolescents [10,11,12,13]. Notably, the risk of SCD does not follow a linear progression with age, as it remains low in patients aged 60 years or older who have demonstrated long-term survival and disease tolerance [14]. Furthermore, no significant differences in SCD risk have been observed based on sex or race [15]. In patients with a risk of 4 to 6%, an ICD may be considered, and in patients with a 5-year risk ≥ 6%, an ICD should be considered [2,3].

Despite the identification of clinical features strongly associated with an increased risk of sudden cardiac death (SCD), this tragic outcome still occurs in patients considered to be low risk [7,16]. This underscores the need for improved risk stratification methods. Preventing SCD is further complicated by the challenges of living with an ICD, which, while lifesaving, can impose significant burdens, including inappropriate shocks, lead malfunctions, infections, and the necessity for pulse generator replacements [17]. These complications can negatively impact patients’ quality of life, leading to psychological distress and higher healthcare utilization. Consequently, an imaging technique or strategy that reliably minimizes ICD implantation in genuinely low-risk patients would offer tremendous clinical value, both by sparing patients from unnecessary interventions and by optimizing healthcare resources.

Late gadolinium enhancement (LGE) on cardiac magnetic resonance (CMR) imaging has been a valuable tool in HCM management [8,18,19]. It reflects intramyocardial fibrosis, a feature directly correlated with the severity and incidence of arrhythmias in HCM. Although LGE is not formally incorporated into the 5-year SCD risk score, it is often used to guide ICD placement decisions [3]. However, LGE has limitations. While it indicates myocardial fibrosis, it is more commonly observed in the thickest left ventricular (LV) segments and may not always align with clinical risk. For instance, extensive LGE is sometimes found in asymptomatic elderly patients with HCM with normal systolic function and no adverse outcomes. Moreover, LGE might not capture early myocardial changes in patients without visible fibrosis, who could still harbor significant risks due to underlying myocardial dysfunction or deformation [6]. These subclinical abnormalities may elevate SCD risk but remain undetectable by LGE.

This prompts an important question: can we identify a more accurate approach to predict SCD in HCM? The answer may lie in unraveling the pathological mechanisms driving ventricular arrhythmias, particularly in cases where CMR does not reveal significant fibrosis. Understanding these mechanisms could enhance risk stratification and potentially lead to better clinical outcomes.

Positron emission tomography (PET) has emerged as a valuable tool for exploring these mechanisms. Using tracers such as O^15^-labeled water or N^13^-labeled ammonia (^13^NH_3_), PET provides detailed imaging of myocardial blood flow (MBF) and coronary flow reserve [20,21,22]. Even in the absence of epicardial coronary artery disease (CAD), patients with HCM often exhibit severely impaired coronary flow reserve, reflecting underlying microvascular dysfunction [20,21,22]. This dysfunction, a hallmark of HCM, is linked to disease severity and progression. Additionally, reduced MBF reserve correlates strongly with myocardial fibrosis detected through CMR, underscoring the role of microvascular dysfunction as a precursor to structural cardiac abnormalities [21,23,24,25,26].

While myocardial fibrosis is a well-known risk factor for arrhythmias and SCD, there is increasing evidence that myocardial cell metabolism may be disrupted in HCM, even before fibrosis becomes evident. 18-fluorodesoxyglucose (FDG) PET imaging has been sporadically investigated in HCM, offering insights into metabolic alterations within the myocardium [27,28]. Intense ^18^F-FDG uptake in HCM suggests a metabolic shift from fatty acid oxidation to glucose metabolism, potentially serving as a marker of early disease stages. Interestingly, FDG uptake is generally low in regions of established fibrosis, as indicated by late gadolinium enhancement (LGE) in CMR [29]. This suggests that heightened FDG uptake may indicate early pathological changes preceding fibrosis, although its prognostic implications remain unclear.

Beyond metabolic imaging, PET tracers targeting fibroblast activation protein inhibitors (FAPIs) offer promise in detecting early-stage myocardial fibrosis. By identifying fibroblast activity, FAPI-PET could reveal structural changes that precede those detectable by CMR, offering a novel intermediate marker of disease progression in HCM [30].

This review aims to synthesize the most relevant data on disease progression and risk stratification using advanced PET imaging techniques in HCM. By integrating insights from PET-derived assessments of perfusion, metabolism, and fibrosis, we seek to highlight the potential of this modality in transforming HCM management and prognosis.

## 2. PET Imaging of Microvascular Disfunction in Hypertrophic Cardiomyopathy

Myocardial perfusion imaging (MPI), performed using SPECT and more recently PET, has shown significant diagnostic and prognostic utility in managing patients with known or suspected coronary artery disease (CAD) [31,32]. By combining radiolabeled tracer imaging with either physiological (exercise) or pharmacological (vasodilator) stress, this approach effectively detects regional myocardial ischemia and infarction, which are often the root causes of patient symptoms [31]. These imaging biomarkers play a critical role in guiding medical and interventional therapies aimed at alleviating symptoms and reducing the risk of major adverse cardiac events, including myocardial infarction (MI), heart failure, cardiac arrest, and stroke [31].

PET imaging, using tracers such as ^13^NH_3_, ^15^O-water, and ^11^C-acetate, has been widely validated in clinical studies for the non-invasive measurement of MBF both at rest and during pharmacologic stress in HCM [20,25,33].

### 2.1. Mechanisms of Microvascular Dysfunction

Myocardial flow reserve (MFR) is the ratio of stress MBF (maximum MBF during exercise of pharmacologic stress) to rest MBF; a ratio > 2.5 is considered normal, whilst a ratio < 2 refers to reduced MFR. In the absence of CAD, impaired MFR is an indicator of coronary microvascular dysfunction (CMD) [23]. CMD in HCM is related to thickening of the arterial wall and luminal narrowing of the intramural coronary microvasculature, associated with a reduced capillary density [34,35]. Also, myocyte disarray, extrinsic compressive forces, and increased metabolic demand in the hypertrophied myocardium lead to impaired MBF and MFR and the development of subendocardial myocardial ischemia in HCM [25,34,35].

### 2.2. Predictors of Microvascular Dysfunction

In HCM, a consistent inverse correlation has been found between MBF, MFR, and the LV maximum wall thickness (MWT) [23,36]. This was earlier highlighted by ex vivo studies, which observed an inverse relationship between coronary arteriolar lumen size and LV hypertrophy in HCM hearts [37,38]. In a case report of a patient with apical HCM (AHCM), global MFR was normal at rest, but exhibited a distinct gradient of decreasing flow reserve from the basal to the apical segments with stress [39]. MBF was found to be higher in patients with non-obstructive HCM, compared to the obstructive variant (OHCM) [23]; however, this was not related to the left ventricular outflow (LVOT) gradient [23]. In contrast, other studies have reported a significant negative correlation between LVOT gradient and MBF and MFR [36,40].

On the other hand, patients with HCM may have variable MFR results at diagnosis, ranging from normal values to severely reduced MFR [22,23,24,26,41]. A possible explanation for this heterogeneity could be the genetic transmission factor, as observed by Olivotto et al., where genotype (sarcomeric)-positive patients with HCM displayed lower stress MBF according to PET and higher LGE according to CMR than genotype-negative patients [21]. This relation was independent of baseline characteristics, such as age, LV ejection fraction (LVEF), maximal LV wall thickness, and LVOT gradient [21].

### 2.3. Clinical Implications

Impaired MFR and ischemia are closely linked to myocardial fibrosis in HCM. Patients with LGE observed using CMR imaging often exhibit impaired global MFR, with fibrotic myocardial segments showing lower stress MBF compared to non-fibrotic segments [25,34,35,41,42]. However, the relationship between ischemia and fibrosis is not entirely straightforward. Fibrosis can occur in approximately 8% of HCM cases with normal MFR, indicating that LGE may exist even in the absence of myocardial ischemia [42,43]. Notably, these patients might carry a lower risk of future adverse events, contrasting with those who have both fibrosis and impaired MFR [42].

This variability underscores the complexity of using LGE as a sole predictor for clinical outcomes. The inconsistent prognostic value of LGE may be partially explained by the presence or absence of concurrent ischemia. Identifying HCM subgroups based on both fibrosis markers and perfusion abnormalities could significantly improve risk stratification. For example, patients with HCM with LGE and concomitant ischemia may be at an elevated risk of adverse events, including SCD. The combination of ischemia, fibrosis, and myocardial disarray might create a highly proarrhythmic substrate that predisposes individuals to lethal arrhythmias [44,45].

Subendocardial ischemia is particularly concerning, as it can result in action potential shortening, dispersion of ventricular repolarization, and the initiation of reentrant arrhythmias [44,45]. However, studies exploring the relationship between impaired MBF and arrhythmic events have shown mixed results. Early research failed to establish a correlation between reduced MBF and the occurrence of syncope or non-sustained ventricular tachycardia (NSVT) on Holter monitoring [46]. More recent studies have found that while global and regional MBF at rest and stress were similar in patients with HCM with and without ventricular tachycardia (VT), a higher MBF heterogeneity index—defined as the ratio of the highest to the lowest regional MBF values—was associated with VT (sustained and non-sustained) [47].

Magnusson et al. [24] further demonstrated that a lower MBF gradient during stress, assessed with ^15^O-water or ^13^N_3_ PET, was significantly associated with NSVT. Additionally, myocardial oxygen consumption (MVO2), measured using ^11^C-Acetate PET, was elevated in patients with HCM with NSVT [24]. These findings suggest a potential link between increased metabolic demand and arrhythmic risk. Patients with NSVT also tended to exhibit more dilated left ventricles (LV) on PET imaging [46].

On the other hand, LV dilation during a vasodilator stress test has been associated with reduced MBF and MVD in patients with HCM [41,48]. This LV dilation, along with a reduction in LVEF during the vasodilator stress test, could result from the redistribution of blood flow from the maximally vasodilated subendocardial region to the subepicardial region. A higher LV outflow tract (LVOT) gradient can impair endocardial hyperemic blood flow due to increased compression of the endocardial layer [48]. Additionally, patients with HCM who exhibit LV dilation detected by PET during vasodilatory stress testing often demonstrate significant LV hypertrophy, more severe diastolic dysfunction, and lower MFR, reflecting a greater degree of LV myopathy [41].

Impaired MFR can contribute to a wide range of clinical presentations and disease progression in HCM, from asymptomatic states to angina, dyspnea, progression to heart failure, and even ventricular arrhythmias (VA) [34,41,42,49]. This variability underscores the utility of PET imaging in quantitatively assessing myocardial perfusion in HCM and detecting CMD, which is linked to several adverse cardiovascular outcomes. However, the role of PET imaging in stratifying the risk of SCD, particularly in identifying VA risk, remains uncertain. Most available data are derived from limited samples and rely on hard outcomes, which makes drawing definitive conclusions challenging.

**Figure 1:**
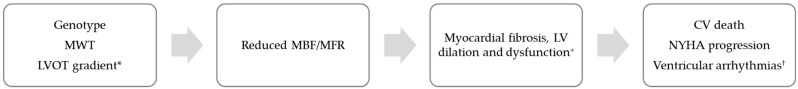
Predictors and outcomes of microvascular dysfunction in HCM. References: * [21,23,36,39]; ^+^ [35,41,42,48]; ^†^ [22,24,49]. Abbreviations: CV: cardiovascular; HCM: hypertrophic cardiomyopathy; LVOT: left ventricular obstruction outflow tract; MBF: myocardial blood flow; MFR: myocardial flow reserve; MWT: maximum wall thickness.

Further investigations are warranted to enhance our understanding of the relationship between PET-derived MBF quantification and myocardial fibrosis assessment in patients with HCM. Such studies could provide critical insights into the mechanisms driving SCD and VA, enabling better risk stratification. PET imaging, with its ability to provide detailed and quantitative measures of perfusion and metabolic alterations, holds promise as a tool for improving prognostic assessment in HCM. Nonetheless, large-scale studies are necessary to establish its definitive role in guiding clinical decisions, particularly regarding SCD prevention and VA risk management. Table 1 summarizes some of the studies that describe MBF and MFR impairment in the presence of several HCM factors. Figure 1 highlights the significant findings, showcasing the predictors and outcomes associated with microvascular dysfunction in HCM.

### 2.4. Limitations

Generally, a reduction in MFR is associated with an increased risk of cardiac mortality [50]. The accurate quantification of MBF requires calculating tracer uptake in the left ventricle and myocardium from imaging data. However, throughout the processes of data acquisition, reconstruction, post-processing, and interpretation, several pitfalls can arise. These pitfalls may compromise the reliability of uptake calculations and, consequently, MBF measurements [50].

In a study where MFR exhibited no discrepancies, MBF showed inconsistencies with narrower limits of agreement [51]. Therefore, it remains unclear whether MBF, MFR, or a combination should be used—and at a global, vascular, or regional level—to achieve optimal prognostic insight [50].

These discrepancies are evident in the assessment of prognosis in HCM, with varying results reported for several disease outcomes (as detailed in Section 2.3 and Table 1).

## 3. ^18^F-FDG-PET Imaging Assessment of Myocardial Metabolism in Hypertrophic Cardiomyopathy

^18^F-FDG-PET is routinely used to diagnose the systemic involvement of malignant tumors or in inflammatory diseases due to their higher glucose metabolism. Increased uptake of ^18^F-FDG -PET in the myocardium may indicate the presence of cardiac tumors or inflammatory disease (such as sarcoidosis) [52,53,54,55]. Abnormal uptake of ^18^F-FDG in HCM has been reported previously [27,28,56,57].

### 3.1. Physiopathology of Myocardial Metabolism

The explanation found for this increased ^18^F-FDG uptake in HCM is caused by increased glucose utilization and an impaired fatty acid metabolism [57]. The main energy source of a normal myocardium is fatty acids; however, in pathologic conditions, such as ischemia or inflammation, there can be a shift in the energy substrate to glucose metabolism. In HCM, there is an increase in the energy demand related to myocardial hypertrophy, inflammatory cell infiltration, and microvascular disfunction that leads to ischemia and consequently to a mismatch in the supply/demand of blood flow [57].

### 3.2. Myocardial Metabolism Patterns

In HCM, increased ^18^F-FDG uptake may exhibit varying patterns depending on the phenotypic expression and stage of disease progression.

An earlier study reported that regional ^18^F-FDG uptake was higher in patients with asymmetrical septal hypertrophy and dilated-phase HCM [28].

LV ^18^F-FDG uptake was also described to be more non-homogeneous in younger patients than in middle-aged to elderly patients and was not associated with non-homogeneity in LV wall thickness [56].

Aoyama et al. [57] compared ^18^F-FDG uptake in obstructive HCM (OHCM) and in non-obstructive HCM (NOHCM). They found that ^18^F-FDG uptake was limited to the hypertrophied myocardium in NOHCM, whereas in OHCM it was extensive and beyond the hypertrophied segments [57]. The authors also found that a reduction in intra-LV obstruction using alcohol septal ablation can affect myocardial metabolic shift in the lateral myocardium of OHCM, with a reduction in FDG uptake in this region [57].

Regarding AHCM, the intensity of space-shaped FDG uptake around the left ventricular apex was found to be associated with the progression of myocardial hypertrophy that was detected with ECG change, apical asynergy, and impaired coronary flow reserve [52]. However, there was no significant relationship between apical wall thickness and the intensity of ^18^F-FDG uptake. As a result, the metabolic change that occurs in AHCM might be associated with pathophysiological but not with morphological change [52]. This increased apical ^18^F-FDG uptake has been described in other case studies [55,58,59], where moderate mononuclear cell aggregation was identified in the myocardial biopsy [58]. Then, increased ^18^F-FDG uptake may reflect inflammation caused by increased glucose utilization in the hypertrophied myocardium.

### 3.3. Myocardial Metabolism and Fibrosis

When associating ^18^F-FDG uptake to LGE, in NOHCM, it was present in hypertrophied segments [57]. This may indicate that the myocardium requiring glucose metabolism also undergoes fibrotic changes. It is possible that in this group of patients, ^18^F-FDG uptake may indicate the risk of the development of fibrosis. In HOCM, ^18^F-FDG was widely distributed in both hypertrophied septal and remote lateral segments, whereas LGE was not detected in those segments [57]. Since LGE reflects progressed myocardial cell damage, whereas ^18^F-FDG uptake reflects metabolic disorders in an early stage of myocardial cell damage, the patients with HOCM in that study might not have developed myocardial fibrosis.

Also, in AHCM, increased ^18^F-FDG uptake was observed in the apical to mid LV myocardium, whereas lower ^18^F-FDG uptake areas matched areas of LGE [58]. In another case report, intense ^18^F-FDG uptake was observed in the LV, but was relatively low in the apex, where LGE by CMR was evident [29].

In one case report, reduced ^18^F-FDG uptake was noted within the hypertrophic septum corresponding to the area of LGE [27]. In another case report, reduced myocardial ^18^F-FDG uptake in the anterior and apical interventricular septum of the LV suggested partially necrotic tissue [60]. If the patient underwent CMR, these necrotic areas would probably correspond to LGE areas.

### 3.4. Clinical Implications

**Figure 2:**
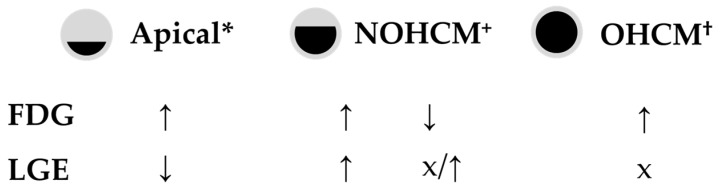
Patterns of ^18^F-FDG uptake and LGE in different types of HCM. References: * [52,55,58,59]; ^+^ [29,57]; ^†^ [57]. Abbreviations: AHCM—apical hypertrophic cardiomyopathy; LGE—late gadolinium enhancement; NOHCM—non-obstructive hypertrophic cardiomyopathy; OHCM—obstructive hypertrophic cardiomyopathy; ↑increase; ↓ decrease; x no effect.

The studies described in this section suggest that (1) ^18^F-FDG uptake is possibly related to the degree of severity of HCM, where a higher uptake can represent a poor prognosis; (2) ^18^F-FDG uptake can be detected in earlier phases of the disease, even before the development of fibrosis; and (3) ^18^F-FDG uptake can represent myocardial areas predisposed to the development of fibrosis. Table 2 and Figure 2 provide a summary of the data on ^18^F-FDG uptake across different stages of HCM.

## 4. Early-Stage Myocardial Fibrosis Assessed by Radiolabeled Fibroblast Activation Protein Inhibitors (FAPIs)

### 4.1. Physiopathology of Fibroblast Activation in HCM

Myocardial fibrosis is a dynamic process driven by the activation of cardiac fibroblasts, leading to a disrupted extracellular matrix homeostasis and excessive collagen deposition. Early-stage myocardial fibrosis is marked by fibroblast activation, characterized by the expression of fibroblast activation protein (FAP). FAP is a type II integral membrane glycoprotein with dipeptidyl peptidase and type I collagenase activity, uniquely associated with activated fibroblasts [61]. Radiolabeled tracers targeting fibroblast activation protein inhibitors (FAPI) have been developed to identify activated fibroblasts and are only extensively utilized in PET/CT imaging for cancer detection [62]. Additionally, studies have reported the use of FAPI PET/CT imaging in patients with myocardial infarction [63] and dilated cardiomyopathy [64].

### 4.2. Distribution of Fibroblast Activation in HCM: Predictors and Prognostic Implications

A case report demonstrated the heterogeneous uptake of ^18^F-FAPI and ^18^F-FDG in AHCM. In FAPI imaging, the highest uptake was observed in the septal and lateral walls, while in FDG imaging, the intense uptake was predominantly localized to the anterior wall [65].

In two recent studies including patients with HCM, FAPI myocardial activity was higher than in healthy controls and was detected in non-hypertrophied myocardial segments [30,66]. FAPI activity was also correlated with the SCD risk score [30] and with left ventricular strain [66]. Importantly, in segments without abnormal findings on CMR tissue characterization, the strain capacity of segments with positive FAPI uptake was lower than that of segments with negative FAPI uptake [66].

## 5. Discussion

This review highlights several studies that underscore the potential of PET in refining prognostic stratification for HCM. MBF and MFR measurements provide valuable insights into microvascular dysfunction, ischemia, and fibrosis in HCM patients [21,23,35,36,39]. These abnormalities are intricately linked to disease severity, and their identification through PET imaging offers a non-invasive means to evaluate disease progression and risk [22,24,41,46,48,49].

Moreover, PET imaging of myocardial metabolism with ^18^F-FDG appears particularly promising. This modality may enable the detection of early disease stages before the onset of fibrosis, ischemia, or microvascular dysfunction. Early metabolic abnormalities, indicated by increased FDG uptake, could reflect subtle myocardial alterations, paving the way for timely intervention [27,29,52,55,56,57,58,59,60]. If validated, ^18^F-FDG PET could emerge as a critical diagnostic and prognostic tool for HCM.

Additionally, novel PET tracers targeting fibroblast activation protein inhibitors (FAPIs) have shown potential for identifying early-stage myocardial fibrosis. These tracers provide a more dynamic and nuanced assessment compared to LGE in CMR. FAPI-PET imaging might serve as an intermediate marker for disease progression, offering a higher sensitivity in detecting early fibrotic changes than traditional imaging modalities [30,65,66].

However, the studies reviewed reveal inconsistencies in the relationship between PET-derived metrics and the prognostic stratification of HCM. These discrepancies emphasize the need for comprehensive and standardized research to fully elucidate PET’s diagnostic and prognostic capabilities.

### Future Directions

PET imaging holds substantial promise in refining the prognostic stratification of HCM, particularly in SCD risk. By identifying parameters such as MBF, MFR, and increased uptake of tracers like ^18^F-FDG and FAPIs, PET could offer a more nuanced understanding of SCD risk. This is especially valuable for cases where traditional risk factors are absent or insufficiently predictive. A clearer identification of high-risk patients through PET imaging could facilitate earlier and more targeted interventions, potentially reducing the incidence of SCD. Conversely, PET’s ability to stratify low-risk patients could spare individuals the physical, psychological, and financial burdens of unnecessary ICD placement.

However, challenges remain. The direct correlation between MBF, FDG uptake, and traditional SCD risk factors requires further investigation to establish PET as a standalone decision-making tool. It is not yet clear whether PET imaging alone could reliably guide ICD implantation or justify avoiding it altogether.

Beyond risk stratification, PET offers potential as a tool for monitoring therapeutic efficacy in HCM. For instance, it could track changes in myocardial perfusion and metabolism following surgical interventions like septal myectomy or alcohol septal ablation. Additionally, PET imaging could assess the impact of innovative treatments, such as myosin inhibitors like mavacamten [67], by evaluating their effects on myocardial function and disease progression. Such applications would not only enhance treatment planning but also provide real-time insights into the effectiveness of novel therapies.

In summary, while PET imaging is a promising frontier in HCM prognosis and management, more robust studies are needed to validate its applications and fully integrate it into clinical practice. Its role in bridging diagnostic gaps and optimizing therapeutic outcomes positions it as a pivotal tool in the evolving landscape of HCM care.

## 6. Conclusions

This review presents valuable insights into the use of PET imaging in HCM, focusing on its ability to assess MBF, myocardial metabolism, and early-stage fibrosis. Abnormal MBF and MFR are linked to various prognostic factors that influence disease progression and severity, including myocardial wall thickness, arrhythmias, myocardial fibrosis, and ventricular dilation. Furthermore, ^18^F-FDG PET imaging emerges as a promising tool for detecting early-stage myocardial metabolic changes, potentially preceding fibrosis and ischemia. The innovative use of FAPI-PET imaging also shows promise in identifying early myocardial fibrosis with greater sensitivity compared to traditional CMR.

However, conflicting findings in the literature underline the need for more robust research to clarify PET’s role in HCM prognosis and risk stratification. If PET imaging can reliably associate specific parameters—such as abnormal MBF, MFR, or increased FDG/FAPI uptake—with SCD risk, it may revolutionize risk prediction, enabling the more precise identification of patients for ICD implantation or potentially avoiding unnecessary interventions.

Additionally, PET imaging holds potential for monitoring therapeutic outcomes, evaluating changes in perfusion and metabolism following interventions such as septal myectomy and ablation, or treatment with myosin inhibitors. These advancements position PET imaging as a versatile and evolving modality for advancing HCM management.

## Figures and Tables

**Table 1:** Studies describing MBF and MFR impairment assessed by PET-CT according to HCM features.

**Predictors of myocardial perfusion impairment in HCM**							
**Author (journal, year)**	**Pts (N)**	**Tracer**	**Predictor**	**MBF**	**MFR**	**Outcome**	**Ref**
Olivotto (JACC, 2011)	61	^13^NH_3_	Genotype	<1.5		LGE	[21]
Knaapen (Am J Physiol Heart Circ Physiol, 2008)	18	^13^NH_3_	MWT	Reduced			[36]
			LVOT gradient	Reduced			
Bravo (J Nucl Med, 2012)	33	^13^NH_3_	MWT	Reduced	Lower in NOHCM		[23]
			LVOT gradient	No correlation	No correlation		
Buchwald (J Nucl Cardiol, 2022)	Case report	^13^NH_3_	MWT (apex)		Reduced (apex)		[39]
**Outcomes resulting from myocardial perfusion impairment in HCM**							
**Author (journal, year)**	**Pts (N)**	**Tracer**	**Predictor**	**MBF**	**MFR**	**Outcome**	**Ref**
Castagnoli (Eur J Nucl Med Mol Immaging, 2016)	100	^13^NH_3_		<1.1		CV death NYHA progression Ischemic stroke VA	[22]
Cechi (N Engl J Med, 2003)	51	^13^NH_3_		Reduced	Reduced	CV death	[49]
Sotgia (J Nucl Med, 2008)	34	^13^NH_3_		Reduced		Fibrosis (LGE)	[35]
Bravo (Circ Cardiovasc Imaging, 2013)	47	^13^NH_3_		Reduced	Reduced	Fibrosis (LGE) (small proportion with normal perfusion)	[42]
Lorenzoni (Eur Heart J, 1997)	84	^13^NH_3 _ H_2_^15^O		No variation	No variation	Syncope NSVT	[46]
Lu (Am J Cardiol, 2018)	133	^13^NH_3_		No variation	No variation	NSVT	[24]
				Lower gradient			
				Higher MVO_2_ consumption			
Bravo (J Nucl Cardiol, 2016)	61	^13^NH_3_		Reduced	Reduced	LV dilation	[41]
Lu (J Nucl Cardiol, 2020)	108	^13^NH_3_			Reduced	LV dilation and dysfunction	[48]

Abbreviations: CV: cardiovascular; HCM: hypertrophic cardiomyopathy; LGE: late gadolinium enhancement; LVOT: left ventricular obstruction outflow tract; MBF: myocardial blood flow; MFR: myocardial flow reserve; MWT: maximum wall thickness; MVO_2_ myocardial oxygen consumption; NSVT: non-sustained ventricular tachycardia; VA; ventricular arrythmia.

**Table 2:** Studies and case reports regarding ^18^F-FDG uptake across different stages of HCM.

Author (Journal, Year)	Pts (N)	18F-FDG Uptake Distribution	18F-FDG Uptake and LGE	Ref
Uehara (Ann Nucl Med, 1998)	32	Higher in dilated-phase HCM Lower in AHCM		[28]
Kagaya (Am J Cardiol, 1992)	16	Younger pts have non-homogeneous uptake		[56]
Aoyama (PLOS one, 2017)	30	NOHCM—limited to the hypertrophied segments OHCM—beyond hypertrophied segments	NOHCM—LGE present in hypertrophied segments OHCM—LGE not commonly observed	[57]
		NOHCM—related to hsTnI levels OHCM—related to BNP levels		
		OHCM—reduced with septal ablation		
Kong (Nucl Med Mol Imaging, 2013)	Case report	Reduced in the asymmetrical hypertrophied septum	Lower uptake matched LGE areas (septum)	[27]
Funabashi (Int J Cardiol, 2006)	Case report	Reduced in the anterior and apical inter-ventricular septum		[60]
Takeishi (J Nucl Cardiol, 2016)	Case report	Increased in middle walls, reduced in apex	Lower uptake matched LGE areas (apex)	[29]
Katagiri (Ann Nucl Med, 2017)	Case series (17)	AHCM—increased in the apex		[52]
Norikane (J Nucl Cardiol, 2019)	Case report	AHCM—increased in the apex		[55]
Yamamoto (Journal of Cardiol Cases, 2017)	Case report	AHCM—increased in the apex	Lower uptake matched LGE areas	[58]
Miyamoto (J Nucl Cardiol, 2022)	Case report	AHCM—increased in the apex		[59]

Abbreviations: AHCM—apical hypertrophic cardiomyopathy; BNP—brain natriuretic peptide; hsTnI—high sensitivity troponin I; LGE—late gadolinium enhancement; NOHCM—non-obstructive hypertrophic cardiomyopathy; OHCM—obstructive hypertrophic cardiomyopathy.

## Data Availability

Not applicable.

## Abbreviations

AHCM	apical hypertrophic cardiomyopathy
BNP	brain natriuretic peptide
CAD	coronary artery disease
CMD	coronary microvascular dysfunction
CMR	cardiac magnetic resonance
CV	cardiovascular
FAPI	fibroblast activation protein inhibitors
FDG	fluorodeoxyglucose
HCM	hypertrophic cardiomyopathy
hsTnI	high sensitivity troponin I
ICD	implantable cardioverter-defibrillator
LGE	late gadolinium enhancement
LVEF	left ventricular ejection fraction
LV	left ventricle
LVOT	left ventricular outflow tract
MBF	myocardial blood flow
MFR	myocardial flow reserve
MI	myocardial infarction
MVO_2_	myocardial oxygen consumption
MWT	maximum wall thickness
NOHCM	non-obstructive hypertrophic cardiomyopathy
NSVT	non-sustained ventricular tachycardia
OHCM	obstructive hypertrophic cardiomyopathy
PET	positron emission tomography
SCD	sudden cardiac death
VA	ventricular arrhthymias
VT	ventricular tachycardia
